# Genetic examination of the putative skull of Jan Kochanowski reveals its female sex

**DOI:** 10.3325/cmj.2011.52.403

**Published:** 2011-06

**Authors:** Tomasz Kupiec, Wojciech Branicki

**Affiliations:** Section of Forensic Genetics, Institute of Forensic Research, Krakow, Poland

## Abstract

We report the results of genetic examination of the putative skull of Jan Kochanowski (1530-1584), a great Polish renaissance poet. The skull was retrieved in 1791 by historian Tadeusz Czacki from the Kochanowski family tomb and became the property of the Czartoryskis Museum in Krakow. An anthropological study in 1926 questioned its male origin, which raised doubts about its authenticity. Our report presents genetic evidence that resolves this dispute.

From the sole tooth we obtained a sufficient amount of DNA to perform the analysis of nuclear markers. The analysis of the sex-informative part of intron 1 in amelogenin, genotyped using AmpFiSTR® NGM PCR Amplification Kit and Powerplex® ESI17 Kit human identification systems, revealed the female origin of the tooth. The female origin was further confirmed by the analysis of a portion of amelogenin intron 2, a microsatellite marker located on the X chromosome, as well as by a lack of signal from Y chromosomal microsatellite markers and the sex-determining region Y marker. Data obtained for two hypervariable regions, HVI and HVII, in mitochondrial DNA showed that mtDNA haplotype was relatively frequent among contemporary Europeans. The analysis of a set of single nucleotide polymorphisms relevant for prediction of the iris color indicated an 87% probability that the woman had hazel or brown eye color.

Identification of human remains is an important area of expert work conducted in legal and forensic laboratories. When dealing with the identification of missing persons, the method of choice is often the analysis of polymorphic DNA markers, which has been proven to be very effective in complex cases of identification of victims of war and mass disasters (1-4). Contemporary genetic methods are sensitive and robust enough to cope with the most problematic samples of ancient DNA and thus may cast light on historical controversies concerning famous personages. Examples of such cases include examination of remains traditionally attributed to the evangelist Luke (5); a supposed son of Louis XVI, King of France, and Marie-Antoinette (6); the legendary outlaw Jesse James (7); Italian poet and scholar Francesco Petrarca (8), the Tsarist Romanov family (9,10); and the great astronomer Nicolaus Copernicus (11). We present an additional case of DNA examination of a historical skull, which was assumed to be that of the greatest Polish Renaissance poet – Jan Kochanowski.

Jan Kochanowski was born in 1530 in the small village of Sycyna in Poland. A brilliant intellectual of that era and a graduate of universities in Krakow and Padua, he is best known as the author of several hundred compositions written in Polish and Latin. Kochanowski also served as a secretary to King Sigmund II Augustus. He most probably died in Lublin (Poland) in 1584 at the age of 54 and was buried in a church in Zwoleń with other members of his family. In 1791, Kochanowski’s skull (Figure 1) was retrieved from the Kochanowski family tomb by historian Tadeusz Czacki. The skull afterwards became the property of the Czartoryskis Museum in Krakow, where it is still kept. A controversy over the authenticity of the skull arose in 1926, when an anthropological study questioned its male origin (12). The aim of this study was to use genetic analysis methods to resolve the dispute concerning the sex of the skull.

**Figure 1:**
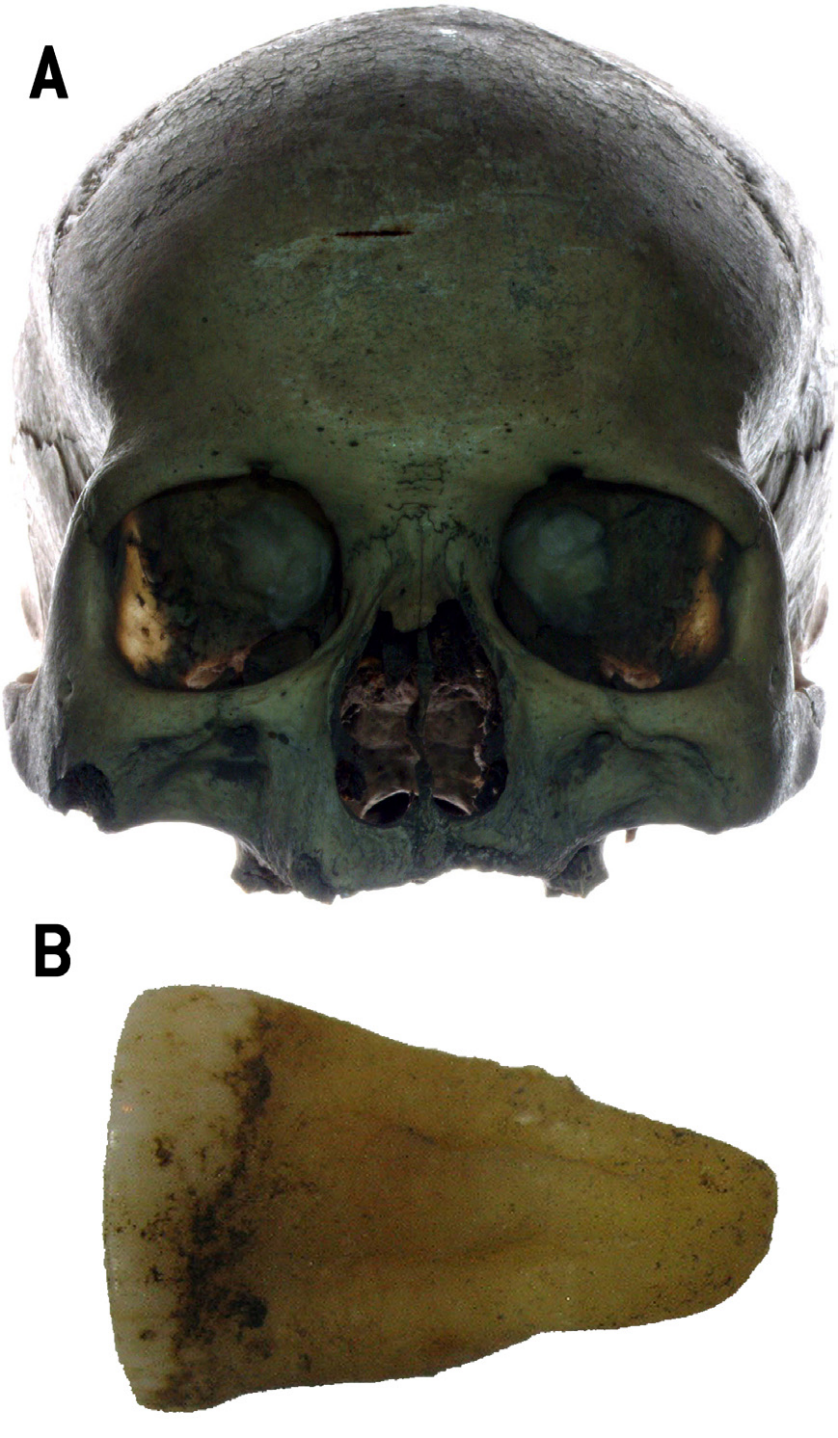
The putative skull of Jan Kochanowski from the collection of the Czartoryskis Museum in Krakow (**A**) and the sole tooth subjected to the analysis (**B**).

## Material and methods

### DNA extraction

The Czartoryskis Museum stipulated that there should be minimal intervention for the purposes of the expert opinion, but agreed that the sole tooth could be used for DNA extraction and genotyping. Before extraction, the tooth was subjected to a purification and decontamination procedure. It was treated with 15% bleach, repeatedly shaken with 70% ethanol and distilled water, and subjected to UV irradiation. It was pulverized using FreezerMill 6750 apparatus (Spex CertiPrep, Metuchen, NJ, USA) and subjected to a standard organic extraction procedure. Approximately 3 g of bone powder was incubated overnight at 56°C with 3mL of buffer composed of 0.5 M EDTA and 10% sodium dodacyl sulfate, with addition of 225 μL proteinase K (10 mg/mL) and 120 μL 1 M DTT. The sample was subjected to double extraction with a mixture of phenol-chloroform-isoamyl alcohol (Sigma-Aldrich, Steinheim, Germany) and concentrated with Amicon Ultra 4 – 30k columns and Microcon 100 (Millipore, Billerica, MA, USA). Negative extraction control was used to check the purity of the used chemicals and consumables.

### DNA quantification and detection of SRY sex informative sequence

The DNA extract was subjected to quantification using the Quantifiler® Human DNA Quantification Kit (Applied Biosystems, Foster City, CA, USA). The Quantifiler® Duo DNA Quantification Kit, which, besides a human-targeted molecular probe, contains a specific probe complementary to the sex-determining region Y (SRY), was used to confirm the female sex of the analyzed skull. Both the analyses were completed using a 7500 Real-Time PCR System, applying the protocols recommended by the manufacturer.

### Genotyping

*Analysis of nuclear polymorphic markers using human identification kits.* Two different human identification kits: AmpFiSTR® NGM (Applied Biosystems) and Powerplex® ESI17 (Promega, Madison, WI, USA) were used to analyze a set of 15 highly variable microsatellite loci and amelogenin sex marker. Both reactions were performed exactly as recommended by the manufacturer’s protocols. Seven microliters (approximately 120 pg) of DNA template was added in both cases. The two kits contain different sets of primers for amplification of the sex-informative segment of amelogenin intron 1, described by Sullivan et al (13), yielding polymerase chain reaction (PCR) products differing in size 6 nucleotides between amelogenin locus on the chromosome X (AMELX) and amelogenin locus on the chromosome Y (AMELY).

*Analysis of amelogenin exon 2/intron 2 sex-differentiation region.* Using primers described by Esteve Codina et al (14), another sex-informative segment of amelogenin was amplified. The analyzed segment is located in intron 2/exon 2 of the amelogenin gene and its amplification gives 55 and 58 bp amplicons for AMELX and AMELY, respectively. The PCR was conducted in a total volume of 12.5 μL. The mixture consisted of 5 μL of amplification mix (150 mM Tris-HCl, 500 mM KCl, pH 8.0, 200 μM dNTPs, and 1.5 mM MgCl_2_), 1.25 μL of PCR primers (200 nM each, forward primer FAM labeled), 0.25 μL of AmpliTaq Gold® DNA Polymerase, and 6 μL of template DNA. The PCR profile was as follows: initial denaturation at 95°C/11-minutes, 28 cycles of (94°C/1 minute, 54°C/1 minute, 72°C/1 minute) and a final elongation at 60°C/45-minutes. PCR products were analyzed on an ABI 3130 XL genetic analyzer using the module for microsatellite analysis and GeneScan 120 LIZ® Size Standard.

*Analysis of Y-STR and X-STR markers*. Seventeen microsatellite markers included in the AmpFiSTR® Yfiler® PCR Amplification Kit (Applied Biosystems) were subjected to the analysis using the standard protocol recommended by the manufacturer. The PCR mixture was composed of 9.2 μL of PCR reaction mix, 5 μL of PCR primers, 0.8 μL of AmpliTaq Gold® DNA Polymerase, and 10 μL of template DNA (approximately 170 pg). Eight microsatellite markers located on the chromosome X were analyzed by the Mentype® Argus X-8 amplification kit (Biotype) using the protocol recommended for biological samples containing a low amount of DNA (34 cycles of amplification). The PCR mixture was composed of 2.5 μL of PCR reaction mix, 1.25 μL of PCR primers, 0.2 μL of Multi Taq2 DNA Polymerase, 4 μL of template DNA (approximately 70 picograms), and water up to 12 μL.

*mtDNA analysis.* Two hypervariable segments in mitochondrial DNA (HVI and HVII) were amplified and sequenced using the previously described primer pairs (L15997-H16236, L16159-H16401, L48-H285, and L172-H408) (15). Amplification was performed in a GenAmp 9700 thermocycler (Applied Biosystems) in a total volume of 10 μL. The PCR reaction mixture consisted of 5 μL of Qiagen multiplex PCR kit (Qiagen, Valencia, CA, USA), 1μL of PCR primers, 1μL of Q solution, and 3 μL of template DNA. The temperature profile was as recommended by the kit manufacturer with an annealing temperature of 58°C (HVI) or 60°C (HVII). The PCR efficiency was checked using the capillary electrophoresis method as implemented in the Qiaxcel system (Qiagen). PCR products were then purified with a mixture of ExoI and SAP enzymes (Fermentas, Glen Burnie, MD, USA). Sequencing reactions were performed using the BigDye Terminator Cycle Sequencing Ready Reaction kit, v.3.1 (Applied Biosystems). The products of sequencing reactions were analyzed on an ABI PRISM 3100 genetic analyzer and SeqScape computer software (Applied Biosystems).

*Prediction of the eye color.* Six single nucleotide polymorphisms located within pigment-related genes, which have been described as the most important eye color predictors (16), were genotyped using multiplex amplification and SNaPshot-based minisequencing. The analyzed SNPs included rs12913832 in *HERC2*, rs1800407 in *OCA2,* rs16891982 in *SLC45A2*, rs12896399 in *SLC24A4*, rs1393350 in *TYR*, and rs12203592 in *IRF4*. Prediction of eye color was based on posterior probabilities for light (defined as blue + green) and dark (defined as hazel + brown) eye colors calculated from the ascertained genotypes and based on their frequency distribution in groups representing four eye color categories, ie, blue, green, hazel, and brown. The probabilities were assessed using the developed Bayesian network implemented in Hugin researcher v. 7.0 computer software (Hugin Expert A/S, Aalborg, Denmark).

## Results and discussion

### Analysis of nuclear markers

The primary aim of this study was to establish the biological sex of the skull. The analysis using the Quantifiler kit indicated that the DNA concentration in the tooth sample was 0.0173 ng/μL, which was sufficient to analyze nuclear markers. DNA was suspended in 70 μL of PCR quality water and approximately 1 ng of DNA was extracted from the tooth. This allowed us to determine the sex of the skull, since this type of analysis can only be made by nuclear DNA markers. If the samples contain a sufficient amount of DNA, they are usually subjected to the analysis of autosomal microsatellite markers, using one of several commercially available human identification kits. Besides the analysis of highly variable short tandem repeat markers (STR), these kits also enable the examination of the fragment of intron 1 in amelogenin that exhibits length polymorphism between two homologous amelogenin loci located on the chromosomes X and Y. This polymorphism has been used for sex determination in forensics since 1993, when it was first described by Sullivan et al (13). Although false female sex designations related to mutations in the AMELY region have been frequently reported, for example in Indians (1.85%) and Sri Lankans (8%), they are rare in Caucasians (0.018%) (17-19).

**Figure 2:**
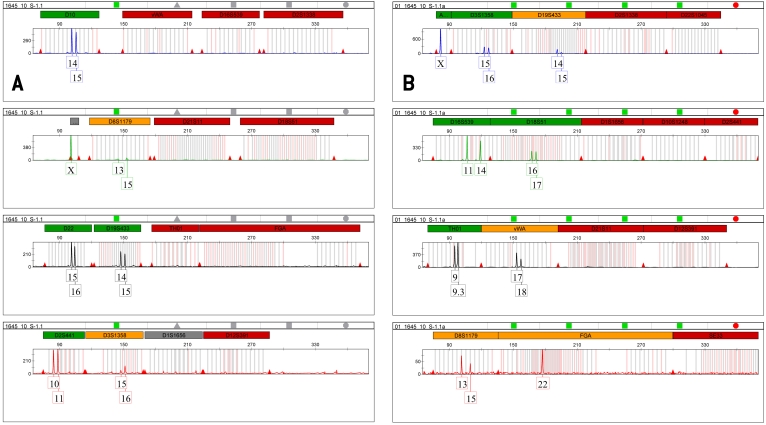
Electropherogram of DNA extracted from the putative tooth of Jan Kochanowski using NGM (**A**) and PowerPlex ESX17 kit (**B**).

**Figure 3:**
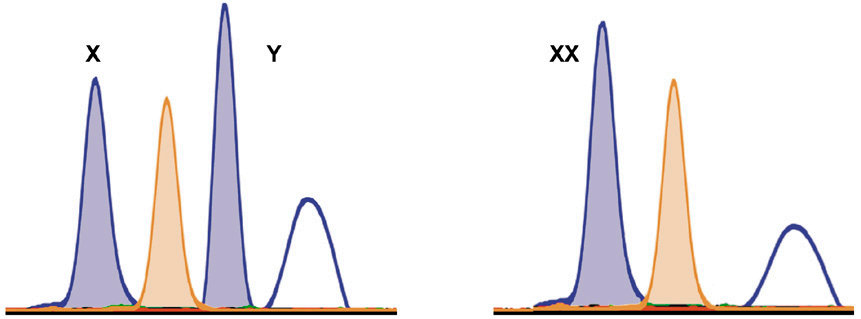
The analysis of amelogenin exon 2/intron 2 length polymorphism: male control (left) and the analyzed tooth (right).

Using two different kits containing different primer sets for amplification of the part of the amelogenin intron 1 locus, NGM and PowerPlex ESX 17, we showed that the tooth belonged to a female person (Table 1 and Figure 2). Despite this, an analysis of additional markers is advisable to exclude the possibility of false designation caused by mutation in the region where amplification primers anneal or by a deletion of the locus or one of its parts. The female sex was confirmed by genotyping of sex-informative length variation in the exon 2/intron 2 segment of the same amelogenin locus, described by Esteve Codina et al (Figure 3) (14). This test is very useful for heavily degraded samples, including old human remains due to the very short amplicon size, 55 bp for AMELX and 58 bp for AMELY. We obtained positive result for DXS10074 marker. Two different alleles (16,18) were ascertained, confirming the presence of two X chromosomes, thus indicating female sex of the skull.

**Table 1:** DNA profiles obtained for the examined tooth sample using two different human identification kits, NGM and PowerPlex ESI 17

Short tandem repeat system	Tooth PowerPlex ESI	Tooth NGM
*D10S1248*	-	14, 15
*vWA*	17, 18	*-*
*D16S539*	11, 14	*-*
*D2S1338*	-	-
*Amelogenina*	X	X
*D8S1179*	13, 15	13, 15
*D21S11*	-	-
*D18S51*	16, 17	*-*
*D22S1045*	-	15, 16
*D19S433*	14, 15	14, 15
*TH01*	9, 9.3	*-*
*FGA*	22	*-*
*D2S441*	-	10, 11
*D3S1358*	15, 16	15, 16
*D1S1656*	-	-
*D12S391*	-	-

The Argus X-8 kit contains the Sullivan primers for amelogenin sex marker. As expected, the analysis of this locus indicated female sex. To exclude the possibility that sex was falsely determined because the complete amelogenin locus had been lost due to deletion of a significant part of the Y chromosome, 17 male-specific Y chromosome markers were subjected to analysis using a Yfiler kit. Using the standard procedure of genotyping, a lack of signal was found, which further supports the hypothesis that the analyzed tooth was of female origin. In humans, SRY gene located originally on the male-specific Y chromosome plays a crucial role in the development of male sex. In very rare cases, translocation of SRY may occur and the male phenotype may be observed in individuals lacking the Y chromosome (14). We excluded this possibility since when we analyzed the sex-determining region Y (SRY) using the Quantifiler Duo kit no signal from SRY was detected.

The performance of both identification kits (NGM and PowerPlex ESX 17) was comparable. Only short amplicon STR markers gave positive amplification results, indicating a significant level of degradation of the analyzed tooth sample. Different configuration in terms of shorter and longer amplicon sizes of particular STR loci and different primer design of NGM and PowerPlex ESX 17 allowed us to obtain data for as many as 11 microsatellite markers (Table 1). These data can be used for a potential future kinship study involving the children and wife of Jan Kochanowski, who are buried in the same tomb, which could finally explain the origin of the analyzed skull.

### Mitochondrial DNA analysis

Since each cell contains many copies of mitochondrial DNA and just 2 copies of nuclear DNA, its analysis is possible even in heavily degraded samples or samples containing minute amounts of DNA (20,21). Even if the quality and quantity of a sample is sufficient for the analysis of nuclear markers, mitochondrial DNA may still remain the only source of data appropriate for complete identification when reference material is limited to samples collected from distant relatives representing the maternal line. Such case was the famous identification of the Tsarist Romanov family, killed by the Bolsheviks in 1918 (9). The analysis of mitochondrial DNA control region HVI and HVII in our study revealed the following differences according to the Cambridge Reference Sequence: 16189C, 16356C, 16362C, 263G, 309.1C, 315.1. A search of the EMPOP database (22) showed that this haplotype was relatively frequent among contemporary Europeans. It was present 18 times in European populations, including Poland, and 3 times outside Europe (USA and Uzbekistan).

### Prediction of iris color

Association studies on human pigmentation, including large genome-wide association studies, have revealed several polymorphisms that together explain a large proportion of the observed variation in iris color and have disclosed that rs12913832 in the *HERC2* gene is a crucial predictor of blue eye color in humans (23-27). For this reason, eye color represents the first visible human trait that can be predicted with high accuracy (16,28,29). We successfully analyzed 5 out of 6 most relevant SNPs for eye color prediction (16). From the ascertained genotype *HERC2*: CT, *SLC45A2*: GG, *SLC24A4*: CC, *TYR*: GA, *IRF4:* CC using Bayesian procedure, we calculated with 87% probability that the woman had dark eye color. Prediction of visible traits from genetic data represents a new area of research and so far its application in real cases has been only sporadically reported (11,30). However, it seems that these analyses will soon gain popularity in forensic investigations (31,32).

### Conclusion

Our report resolved an almost 100-year long dispute on the skull historically attributed to the great Polish poet Jan Kochanowski, providing various genetic evidence that the skull had a female origin and cannot be that of Jan Kochanowski. Genetic data obtained for microsatellite markers and mitochondrial DNA can potentially be used in future identification studies of the skull. Our examination of 7 SNPs representing the most important known human eye color predictors showed with 87% probability that the woman had dark eye color. According to Jan Kochanowski’s writings, his wife had dark eyes. Also, since the woman was approximately 50 years old, as established by anthropological examinations (data not present), and since she was buried in the same tomb, we can speculate that Tadeusz Czacki had retrieved the skull of Kochanowski’s wife Dorota instead of that of Kochanovski. This hypothesis needs further investigation involving anthropological and genetic examination of the skeletons from the Kochanowski family tomb in Zwoleń.
